# Seasonal variation in effects of herbivory on foliar nitrogen of a threatened conifer

**DOI:** 10.1093/aobpla/plx007

**Published:** 2017-02-28

**Authors:** Robert N. Schaeffer, Nicole E. Soltis, Jennifer L. Martin, Aden L. Brown, Sara Gómez, Evan L. Preisser, Colin M. Orians

**Affiliations:** 1 Department of Biology, Tufts University, Medford, MA 02155, USA; 2 Department of Biological Sciences, University of Rhode Island, Kingston, RI 02881, USA; 3 Present address: Department of Entomology, Washington State University, Pullman, WA 99164, USA; 4 Present address: Department of Plant Sciences, University of California Davis, Davis, CA 95616, USA

**Keywords:** *Adelges tsugae*, nitrogen remobilization, nitrogen storage, protein, *Tsuga canadensis*

## Abstract

Invasive herbivores can dramatically impact the nitrogen (N) economy of native hosts. In deciduous species, most N is stored in stem tissues, while in evergreen conifer species N is stored in needles, making them potentially more vulnerable to herbivory. In eastern forests of the USA, the long-lived, foundational conifer eastern hemlock (*Tsuga canadensis*) is under the threat of extirpation by the invasive hemlock woolly adelgid (HWA: *Adelges tsugae*). We assessed the impact of HWA infestation on the patterns of seasonal foliar N availability in hemlock planted in a deciduous forest understory. Over the course of a year, we sampled needles and twigs and measured N, carbon (C), C:N ratio, and total protein concentrations. Tissue sampling events were timed to coincide with key life-history transitions for HWA to determine the association between HWA development and feeding with these foliar nutrients. In uninfested trees, needle and twig N concentrations fluctuated across seasons, indicating the potential importance of N storage and remobilization for the N economy of eastern hemlock. Although N levels in HWA-infested trees also cycled annually, the degree to which N concentrations fluctuated seasonally in tissues was significantly affected by HWA feeding. These fluctuations exceeded N levels observed in control trees and coincided with HWA feeding. HWA feeding generally increased N concentrations but did not affect protein levels, suggesting that changes in N do not occur via adelgid-induced protein breakdown. Herbivore-induced mobilization of N to feeding sites and its rapid depletion may be a significant contributor to eastern hemlock mortality in US forests.

## Teaser

Invasive herbivores can dramatically impact the nitrogen (N) economy of native hosts. In eastern forests of the United States, the foundational conifer eastern hemlock (Tsuga canadensis) is under threat of extirpation by the invasive hemlock woolly adelgid (HWA: Adelges tsugae). Our study suggests that HWA-induced mobilization of N to local feeding sites and its rapid depletion may be a significant contributor to eastern hemlock mortality in U.S. forests.

## Introduction

Nitrogen (N) utilized by trees for growth can be derived from both external and internal sources. Through fertilization (Tripler *et al.* 2006, LeBauer and Treseder 2008), mineralization of soil organic matter (Reich *et al.* 1997), atmospheric deposition (Nadelhoffer *et al.* 1999, Thomas *et al.* 2010), or internal resorption (Millard and Grelet 2010), increases in N availability can have a profound effect on tree growth, highlighting its limiting nature (Rennenberg *et al.* 2009). Internal storage and seasonal redistribution can also be a critical component of the N economy of trees (Millard and Grelet 2010). While deciduous trees store N in woody tissues, often in the form of bark storage proteins (Cooke and Weih 2005), evergreen conifers store N in their foliage primarily in the form of photosynthetic proteins such as ribulose bisphosphate carboxylase (RuBisCo) (Camm 1993, Millard *et al.* 2007). For evergreen conifers, seasonal cycling of N among needles is a critical component of their N economy (Nambiar and Fife 1987, Millard and Proe 1992; Millard and Grelet 2010). For instance, remobilized N has been estimated to provide anywhere from 30 to 60% of the N incorporated into new growth (Rapp *et al.* 1979, Miller 1984, Nambiar and Fife 1987), highlighting the importance of N recycling. High levels of herbivory can often compromise future plant growth; in conifers, this impact may be partially due to the loss of valuable N stored in needles (Honkanen *et al.* 1999, Millard *et al.* 2001) or because of host manipulation by herbivores; many insects cause the accumulation of N at the site of feeding (Girousse *et al.* 2005, Goggin 2007).

Evergreen conifers primarily store N in their youngest needles, and the high nutritional value of newly-produced plant tissues makes them especially prone to attack by both chewing and sucking herbivores (Coley 1980, Crawley 1983, Raupp and Denno 1983). For example, simulated browse of 1-year old *Pinus sylvestris* needles reduced new needle growth in spring (Honkanen *et al.* 1999) and N remobilization by 50% (Millard *et al.* 2001). While eastern hemlock is browsed by white-tailed deer (*Odocoileus virginianus*), whose impact can reduce growth and increase mortality (Stockeler *et al.* 1957, Frelich and Lorimer 1985, Mladenoff and Stearns 1993), this tree faces a more serious threat to its future.

Throughout the eastern USA, eastern hemlock is experiencing significant declines in the wake of invasion by the hemlock wooly adelgid (HWA; *Adelges tsugae*). Introduced to the USA from Japan in 1950 (Souto *et al.* 1996), this exotic hemipteran feeds by inserting its stylet into xylem cells at the base of hemlock needles (Young *et al.* 1995). Lacking natural predators, HWA has spread rapidly throughout the eastern USA and can cause hemlock mortality in as little as 4 years (McClure 1991). Adelgid numbers can rapidly increase in an invaded area, as they undergo multiple generations a year, and easily disperse through wind, wildlife, and human activity such as logging (McClure 1989, 1990). Their lifecycle in the invaded range consists of two parthenogenetic stages on hemlock per year, a rapidly-developing spring generation and an overwintering generation with a longer development period (Ward *et al.* 2004). The April–June progrediens generation emerges in early spring and settles on hemlock foliage produced in the previous growing season. Females in this generation can produce ∼75 eggs per individual and their offspring (sistens generation; July–April) settle on newly produced foliage. Once settled, they enter aestivation until late autumn, after which individuals feed throughout the winter and are capable of producing anywhere from 70 to 200 eggs per female (Paradis 2011). Understanding the seasonal dynamics of nitrogen availability and herbivore activity may elucidate the mechanisms driving hemlock decline and mortality.

Relatively, few studies have investigated the effects of sap-feeding herbivores on woody species (reviewed in Zvereva *et al.* 2010). Conifers may be especially susceptible to sap-feeders because they allocate more storage compounds to foliage than do deciduous trees (Chapin *et al.* 1990). Nutrient storage in the foliage may make conifers more likely to succumb to intense sap-feeding events (Fernandes 1990, Furuta and Aloo 1994, Paine 2000), as temporary nitrogen depletion has the potential to adversely affect tree health over short timescales, particularly during the periods of rapid growth. Indeed, the limited research on the effects of sap-feeders on woody species has revealed that they can negatively affect photosynthesis, growth and reproduction (Zvereva *et al.* 2010), and in certain contexts may even surpass defoliators in their impact on photoassimilates (Llewellyn 1972, Schowalter *et al.* 1981). Given their oft-sessile nature, the survival and fecundity of specialist sap-feeding herbivores depends on local resource availability in their plant hosts. As a result, the timing of herbivore development relative to fluctuations in host nitrogen is critical for insect survival and fecundity (Mattson 1980). As a sessile hemipteran, HWA is also dependent on high-quality local resources during active feeding periods; elevated N levels are found at HWA feeding sites (Gómez *et al.* 2012, Soltis *et al.* 2015).

Previous common garden studies suggest that the impacts of HWA feeding on N dynamics are dependent upon factors that vary over time. Foliage from artificially-infested trees had a lower N concentration than uninfested foliage 5 to 10 months after initial infestation (Miller-Pierce *et al.* 2010). One year later, however, no HWA effect on foliar N was observed. In a separate study using experimental samples collected in the spring, newly-produced HWA-infested foliage had a higher amino acid content than uninfested foliage after one year of infestation (Gómez *et al.* 2012). The amino acid content of foliage from trees infested for 3 years, however, did not differ from control foliage. The changes to N dynamics across multiple years of data collection suggest that the effect of HWA on foliar N varies throughout the progression of infestation. While free amino acids are one of many components making up total N, these studies together suggest a drop in N or a spike in amino acids in the first year of infestation, followed by recovery to levels comparable to uninfested trees. Further, the contrasting results between total N and amino acid dynamics may be due to seasonal variation in the effects of infestation. It is thus still unclear how and when HWA may be causing N resource depletion.

We examined seasonal variation in foliar N availability in eastern hemlocks grown in an understory common garden to 1) assess baseline N dynamics in healthy trees; and 2) understand how these dynamics are affected by adelgid feeding. We examined tissue-level concentrations of N, carbon (C), C:N, and protein as simple indicators of bulk N dynamics. By measuring these indicators over the course of a year, we aimed to clarify previous results indicating a time-dependent HWA effect.

## Methods

### Experimental design

We collected a one-year time course of samples from seedlings in an understory common garden established at the Kingston Wildlife Research Station (Kingston, RI). One-year-old seedlings were purchased from Van Pines Nursery (West Olive, MI, USA) and planted in a grid in April 2011. The seedlings were inoculated with HWA-infested foliage following standard protocols (Butin *et al.* 2007) over the course of three growing seasons, in April 2011, 2012, and 2013. Control trees received a sham treatment using uninfested hemlock foliage to control for tree handling. Seedlings were artificially infested in a randomized complete block design, such that 15 trees each were allocated to HWA and control treatments. All seedlings were caged in wire and netting to prevent deer herbivory and HWA dispersal (Agribon-15, Johnny’s Selected Seeds, Waterville, ME, USA; 90% light transmission). On our first sample date, the seedlings were < 1m in height, approximately 2-years old, with 1 year of HWA infestation in the common garden. Complete details of the common garden are available elsewhere (Gonda-King *et al.* 2014).

### Adelgid abundance

To assess infestation levels, HWA densities were monitored in late fall (October 2012) and early spring (April 2013). Briefly, in each season, two 5 cm branchlets were selected and HWA individuals were counted.

### Tissue sampling and analysis

**Figure 1.**
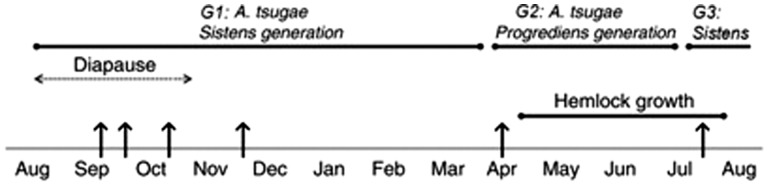
Life cycle of *Adelges tsugae* in the eastern USA in relation to *Tsuga canadensis* seasonal growth. Vertical arrows indicate dates of tissue sampling to assess effects of *A. tsugae* on the nitrogen economy of eastern hemlock. Adapted from Gonda-King *et al.* (2014).

Our time course included a total of seven sampling events from fall 2012 to summer 2013, in an effort to characterize N dynamics over 1 year in the phenology and life history of three HWA generations (G) (Fig. 1). These included: HWA G1 mid-diapause (September 24), HWA G1 immediately preceding diapause break (October 8), HWA G1 newly active (October 31), HWA G1 maturing and active (November 26), HWA G2 newly hatched (April 6), HWA G1 of year 2 newly hatched and preparing to enter diapause (July 8) (Fig. 1). We sampled young (2012) and mature (2011) foliage (twigs and attached needles) for all analyses. In July, we sampled only the newest (2013) elongated twigs and young (2012) twigs; mature (2011) twigs had already lost most needles. Sampled branches were haphazardly selected from the top third of the tree, and the same branches were sub-sampled for each foliar age class. We collected one pooled sample (> 10g of foliage from one to three branch segments with attached needles) per tree per age class at each sampling event. Immediately following these measurements, samples were oven-dried at 75 °C to constant mass. Needle samples were finely ground in a KLECO ball mill (Garcia Machine, Visalia, CA, USA), and twigs were finely ground in a Wiley mill (Thomas Scientific, Swedesboro, NJ, USA). Ground samples were stored desiccated in sealed vials prior to analysis. We determined %C and %N of 5 ± 0.5 mg subsamples by dry combustion on a CHNOS analyzer (vario Micro cube, Elementar Americas, Mt. Laurel, NJ, USA). Finally, protein concentration of tissues was analyzed using 10 ± 0.5 mg subsamples by Bio-Rad Bradford assay (Bradford 1976).

### Statistical analyses

We performed all analyses in R v. 3.2.2 (R Core Team 2015). Linear mixed-effects models (LMMs) were fit using packages *lme4* (Bates *et al.* 2014) and *lmerTest* (Kuznetsorva *et al.* 2013) for analyses of N(%), C(%), C:N, and protein data. The package *lmerTest* uses algorithms of SAS Proc Mixed (Littell *et al.* 1996) to both assess the significance of fixed effects using Type III sums-of-squares and estimate denominator degrees of freedom using Satterthwaite’s approximation. For measures on mature (2011) and young (2012) foliage from September 24 to April 6, we fit LMMs with sample date (quantitative), insect treatment (HWA or control), tissue age (mature or young), tissue type (needles or twig), as well as all possible interactions as factors. Tree identity was included as a random effect. We then proceeded with a backward model selection procedure using the *step* function, where terms are removed sequentially based on having the lowest statistical significance (*P *> 0.1). Protein concentration data for Oct 31 were not included in the statistical analysis due to sample loss.

For analyses of N(%), C(%), C:N, and protein in newest (2013) foliage (sampled only on July 8), we fit separate LMMs with factors insect treatment and tissue type, and their interaction, with tree identity as a random effect. The same resource measures taken on young (2012) foliage on July 8 were included in this analysis for comparison. When appropriate, data were transformed to meet the assumptions of normality and homoscedasticity. Results from these best-fit models [**see Supporting Information— Tables S1 and S2**] are presented.

To compare HWA densities in the fall and spring, we used a Wilcoxon Signed-Rank test.

## Results

### Seasonal N availability in eastern hemlock

**Figure 2.**
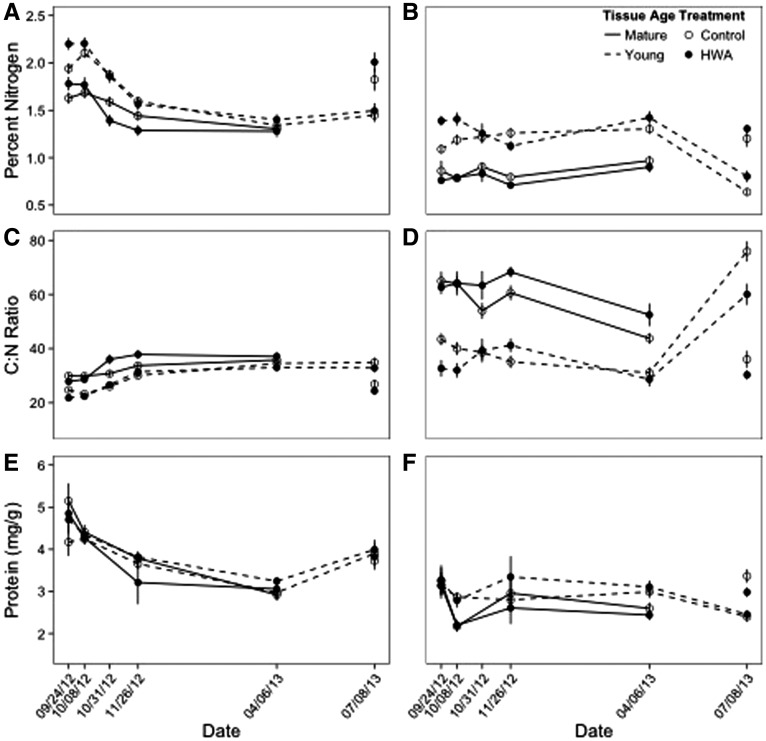
Percent nitrogen (A,B), carbon:nitrogen ratio (C,D), and protein (mg/g) concentration (E,F) of new, young, and mature needles (left-hand panels) and twigs (right-hand panels) from uninfested (open dots) or *Adelges tsugae* (HWA: hemlock woolly adelgid) – infested (closed black dots) *Tsuga canadensis* seedlings. Samples are coded by age as year of growth initiation (Mature: 2011 = solid lines; Young: 2012 = dashed lines). Unconnected dots represent New (2013) growth. Aestivation occurs from July to October.

Nitrogen levels in needles of uninfested trees cycled annually (Fig. 2A). In the fall (September 24), N concentrations in young (2012) and mature (2011) needles averaged 1.94% (±0.05%) and 1.63% (±0.06%) of needle biomass respectively. These levels however decreased by 31% and 20% over the winter, reaching averages of 1.34% (±0.03%) and 1.3 % (±0.05%) as these tissues aged leading into spring (April 6). New growth the following year was similar to September 24 levels. Mirroring N concentrations, protein levels of needles and twigs exhibited significant seasonal cycling (Fig. 2E and F:*F*_3,390.10 _=_ _18.48, *P *< 0.001). Twig N also cycled in an annual fashion (Fig. 2B), though to a less pronounced degree and in an opposite direction.

### Effects of hemlock woolly adelgid on seasonal N availability

Adelgid densities significantly differed between the fall and spring of 2011 and 2012 respectively (Wilcoxon Signed-Rank test: *P *= 0.001). Sistens-generation HWA were five times as dense in the fall (Mean ± SE: 1.67 ± 0.38 insects/cm) compared to the spring (0.32 ± 0.16 insects/cm).

Adelgid feeding affected N concentrations of needle and twig tissues produced in 2011 and 2012 (Fig. 2A and B); however, these effects depended on both the age of tissue examined (*F*_1,523.09 _=_ _16.63, *P *< 0.001), as well as the season in which tissues were sampled (*F*_1,522.09 _=_ _4.29, *P *= 0.04). For instance, HWA infestation elevated the N concentration of young (2012) needles sampled September 24 by 13% relative to controls. Though initially elevated, N in these needles decreased over winter as sistens emerged from aestivation in late fall and began to feed, dropping 36% to an average 1.4 % (±0.06%) in April: a level no different from controls (*post hoc* Tukey HSD test: *P *> 0.05). Moreover, new foliage produced in 2013 following feeding by progrediens on young (2012) and mature (2011) tissues was found to be 11% higher in N concentration than foliage produced by controls, although this effect was only marginally significant (*F*_1,28 _=_ _3.80, *P *= 0.06). Nitrogen dynamics in twigs mirrored that of needles. Young (2012) twigs sampled in September had N levels 28% higher than controls. As sistens began to feed, these levels dropped 19%, to a level 11% lower than controls at the end of November. Interestingly, as sistens continued feeding over winter, N levels increased steadily in HWA-infested twigs to a level 9 % higher than controls in April. Finally, despite affecting N, HWA infestation had no effect on protein levels of tissues between September and April (HWA main effect: *F*_1,29.26 _=_ _0.04, *P *= 0.83) or in July (HWA main effect: *F*_1,27.52 _=_ _0.07, *P *= 0.80).

Adelgid effects on C:N ratios from fall to spring varied according to tissue type and when tissues were examined (Treatment x Tissue x Time interaction: *F*_1,519.74 _=_ _9.27, *P *= 0.002). This effect was largely driven by sistens feeding during winter and resulting shifts in C:N ratios of mature (2011) needles and twigs (Fig. 2C and D). After spring, C:N ratios of tissues produced in 2013 were significantly affected following progrediens feeding (*F*_1,28 _=_ _4.89, *P *= 0.04). Carbon-to-nitrogen ratios were lower for HWA-infested tissues, driven by aforementioned increases in N, rather than changes in C (HWA main effect: *F*_1,28 _=_ _0.0002, *P *= 0.99).

## Discussion

Nitrogen concentrations of tissues fluctuated across seasons in a dynamic manner. The nature of these fluctuations varied across tissue types however, and were significantly affected by HWA feeding. Specifically, we observed high N levels at HWA feeding sites and then a rapid depletion of this resource across seasons as feeding commenced. This suggests that HWA may act as sinks for remobilized N and that changes in N economy is likely a significant contributor to eastern hemlock mortality in North American forests.

### Seasonal variation in N availability in eastern hemlock

Nitrogen concentrations in needles, primary tissues for N storage and utilization in conifers, fluctuated over the course of the year by up to 36%. These fluctuations exhibited an annual cycle, indicative of potential movement of N between storage and actively growing tissues across seasons. Overall, N levels were relatively high in needles in the fall, and then steadily declined until April the following year, as N likely turned over and translocated to new, expanding needles in spring. Though N concentrations are elevated in needles, differences between tissues of different age were observed. The highest levels of N were observed in current year needles (2012) in the fall, while older needles produced in 2011 were comparatively lower. This pattern is consistent with findings for other conifers (Millard and Grelet 2010), highlighting how the bulk of a host’s N is stored in the youngest age class of needle. Our data on patterns of protein concentrations suggest that protein storage and remobilization likely features as part of the hemlock’s N economy as well. Protein levels decreased during fall and winter in current year (2012) needles, with protein levels spiking again in July in maturing 2013 foliage. These losses have been shown in another conifer *Pinus sylvestris*, and also correlate with reduced RuBisCo activity over this period (Gezelius and Hallén 1980, Gezelius *et al.* 1981), as RuBisCo is likely hydrolyzed and resulting N is translocated to other parts of the tree during this period of shoot elongation (Fife and Nambiar 1982, Näsholm and Ericsson 1990).

Nitrogen concentrations in twigs fluctuated in an annual cycle similar to needles, highlighting a likely important role for these tissues in N storage and mobilization. Though both cycled, the patterns and levels of N were in stark contrast, reflecting the inherent differences between these tissues in their chemistry, structure and functionality. This turnover, by upwards of 22%, is driven by slight, but steady increases in N concentration in twigs over winter until spring, when N concentration declines steeply, as observed in young (2012) twigs. Concurrent with losses of N in needles, this pattern may capture N remobilization and translocation, if N from previous-year growth is allocated to branch elongation and new needle expansion in the early summer (Millard and Proe 1992).

### Effects of hemlock woolly adelgid on seasonal N availability

Although adelgid feeding altered N concentrations, the degree and direction of the impact varied according to tissue age, sampling date, and coincided with key life-history transitions over the course of the HWA life cycle (Fig. 1). In early fall (September), HWA sistens produced in the summer are settled on young (2012) and mature (2011) tissues. These tissues are significantly elevated in N relative to control trees, following feeding by the progrediens generation the previous spring. Once sistens emerge from aestivation (late October and November) and begin to actively feed, N concentrations in young (2012) and mature (2011) needles and twigs drops considerably as sistens develop and invest in production of the next generation (progrediens) that will emerge the following spring (April). Though sistens depress N as they feed, once hatched in April, spring-generation HWA (progrediens) commence feeding immediately on young (2012) twigs, enhancing N local to feeding sites. Moreover, this enhancement of N locally can affect N content of the newly produced foliage (2013 growth). Adelgid feeding marginally increased N levels in the newest (2013) tissues relative to controls. This finding supports previous work showing elevated concentrations of free amino acids in uninfested, newly-produced foliage (Gómez *et al.* 2012). Finally, in July, summer-generation larvae (sistens) produced by progrediens settle onto the newest (2013) twigs and enter aestivation, repeating the cycle.

There are several potential explanations for elevated N concentrations in HWA-attacked tissues. First, increased N concentration in HWA-infested tissues may indicate that HWA can act as a strong sink for N. Previous research using measures of N and stable isotopes has indicated the potential for HWA to increase N local to feeding sites (Gonda-King *et al.* 2014, Soltis *et al.* 2015). Aphids and other sap-feeding hemipterans have been similarly shown to increase N transport to feeding sites (Girousse *et al.* 2005, Goggin 2007). Second, following infestation by HWA, eastern hemlock trees often exhibit a reduction in new growth in spring (Preisser and Elkinton 2008; Miller-Pierce *et al.* 2010). Relative to controls, elevated N in new foliage produced in 2013 may merely reflect a concentration effect if both uninfested and infested trees allocate the same amount of N to new growth. Lower levels of N in controls would reflect a dilution effect as foliage expands.

Adelgid feeding had no effect on protein concentrations in eastern hemlock foliage. These results are in support of previous findings that utilized the same assay (Soltis *et al.* 2015), suggesting that HWA feeding does not accelerate protein breakdown in infested tissues. However, our methods for measuring proteins may limit our ability to detect effects or draw conclusions on how proteins are fully impacted by HWA. The Bradford assay is highly specific to proteins with molecular weights in excess of 3 kDA, particularly with arginine, basic, or aromatic amino acid residues. Shorter polypeptides, other nitrogen-containing molecules, and less soluble proteins are not detected (Jones *et al.* 1989). Other forms of N in the needles, including free amino acids, short polypeptides, nucleic acids, secondary metabolites, and inorganic N, were detected in our total-N combustion, but not by the Bradford assay. If HWA are primarily feeding on free amino acids or short, soluble polypeptides available in the xylem ray parenchyma, the lack of effect on proteins as detected by the Bradford assay is perhaps not surprising.

In summary, our data demonstrate seasonal variation in impacts of an invasive herbivore on foliar N of a threatened host. Akin to other evergreen conifers, internal cycling of N for growth is likely an important component of the overall N economy for eastern hemlock. However, winter and spring feeding by HWA sistens and progrediens generations respectively may limit remobilization and transport to new tissues. These findings highlight the significant, but understudied impact that sap-feeders can exert on N of hosts. Future work should clarify how HWA impacts on total N pools and fluxes scale to affect entire forested systems.

## Supplementary Material

Supplementary Data

## References

[plx007-B1] Bates D, Maechler M, Bolker B, Walker S. 2014 *lme4:* Linear mixed-effects models using Eigen and S4.

[plx007-B2] Bradford MM. 1976 A rapid and sensitive method for the quantitation of microgram quantities of protein utilizing the principle of protein-dye binding. Analytical Biochemistry 72:248–254. 942051 10.1016/0003-2697(76)90527-3

[plx007-B3] Butin E, Preisser E, Elkinton J. 2007 Factors affecting settlement rate of the hemlock woolly adelgid, *Adelges tsugae*, on eastern hemlock, *Tsuga canadensis*. Agricultural and Forest Entomology 9:215–219.

[plx007-B4] Camm E. 1993 Photosynthetic responses in developing and year-old Douglas-fir needles during new shoot development. Trees 8:61–66.

[plx007-B5] Chapin FS, Schulze ED, Mooney HA. 1990 The ecology and economics of storage in plants. Annual Review of Ecology and Systematics 21:423–447.

[plx007-B6] Coley PD. 1980 Effects of leaf age and plant life history patterns on herbivory. Nature 284:545–546.

[plx007-B7] Cooke JEK, Weih M. 2005 Nitrogen storage and seasonal nitrogen cycling in *Populus*: bridging molecular physiology and ecophysiology. New Phytologist 167:19–30. 15948826 10.1111/j.1469-8137.2005.01451.x

[plx007-B8] Crawley MJ. 1983. Herbivory: dynamics of plant-animal interactions. University of California Press, Berkeley, CA.

[plx007-B9] Fife DN, Nambiar EKS. 1982 Accumulation and retranslocation of mineral nutrients in developing needles in relation to seasonal growth of young Radiata pine trees. Annals of Botany 50:817–829.

[plx007-B10] Frelich LE, Lorimer CG. 1985 Current and predicted long-term effects of deer browsing in hemlock forests in Michigan, USA. Biological Conservation 34:99–120.

[plx007-B201] Furuta K, Aloo IK. 1994 Between-tree distance and spread of the Sakhalin fir aphid (Cinara todocola Inouye) (Hom., Aphididae) within a population. Journal of Applied Entomology 117:64–71.

[plx007-B11] Gezelius K, Ericsson A, Hällgren J-E, Brunes L. 1981 Effects of bud removal in Scots pine (*Pinus silvestris*) seedlings. Physiologia Plantarum 51:181–188.

[plx007-B12] Gezelius K, Hallén M. 1980 Seasonal variation in ribulose bisphosphate carboxylase activity in *Pinus silvestris*. Physiologia Plantarum 48:88–98.

[plx007-B13] Girousse C, Moulia B, Silk W, Bonnemain J-L. 2005 Aphid infestation causes different changes in carbon and nitrogen allocation in Alfalfa stems as well as different inhibitions of longitudinal and radial expansion. Plant Physiology 137:1474–1484. 15778456 10.1104/pp.104.057430 PMC1088336

[plx007-B14] Goggin FL. 2007 Plant–aphid interactions: molecular and ecological perspectives. Current Opinion in Plant Biology 10:399–408. 17652010 10.1016/j.pbi.2007.06.004

[plx007-B15] Gómez S, Orians CM, Preisser EL. 2012 Exotic herbivores on a shared native host: tissue quality after individual, simultaneous, and sequential attack. Oecologia 169:1015–1024. 22311255 10.1007/s00442-012-2267-2

[plx007-B16] Gonda-King L, Gómez S, Martin JL, Orians CM, Preisser EL. 2014 Tree responses to an invasive sap-feeding insect. Plant Ecology 215:297–304.

[plx007-B17] Honkanen T, Haukioja E, Kitunen V. 1999 Responses of *Pinus sylvestris* branches to simulated herbivory are modified by tree sink-source dynamics and by external resources. Functional Ecology 13:126–140.

[plx007-B18] Jones CG, Hare JD, Compton SJ. 1989 Measuring plant protein with the Bradford assay. Journal of Chemical Ecology 15:979–992. 24271900 10.1007/BF01015193

[plx007-B19] Kuznetsorva A, Brockhoff PB, Christensen RHB. 2013 *lmerTest:* Tests for random and fixed effects for linear mixed effect models (lmer objects of lme4 package).

[plx007-B20] LeBauer DS, Treseder KK. 2008 Nitrogen limitation of pet primary productivity in terrestrial ecosystems is globally distributed. Ecology 89:371–379. 18409427 10.1890/06-2057.1

[plx007-B21] Littell RC, Milliken GA, Stroup WW, Wolfinger RD. 1996 SAS system for mixed models. Cary, North Carolina: SAS Institute.

[plx007-B22] Llewellyn M. 1972 Effects of lime aphid *Eucallipterus tiliae* (Aphididae) on growth of lime *Tilia* x *vulgarius* Hayne. 1. Energy requirements of aphid populations. Journal of Applied Ecology 9:261–282.

[plx007-B23] Mattson WJ. 1980 Herbivory in relation to plant nitrogen content. Annual Review of Ecology and Systematics 11:119–161.

[plx007-B24] McClure MS. 1989 Evidence of a polymorphic life cycle in the hemlock woolly adelgid, *Adelges tsugae* (Homoptera: Adelgidae). Annals of the Entomological Society of America 82:50–54.

[plx007-B25] McClure MS. 1990 Role of wind, birds, deer, and humans in the dispersal of hemlock woolly adelgid (Homoptera: Adelgidae). Environmental Entomology 19:36–43.

[plx007-B26] McClure MS. 1991 Density-dependent feedback and population cycles in *Adelges tsugae* (Homoptera: Adelgidae) on *Tsuga canadensis*. Environmental Entomology 20:258–264.

[plx007-B27] Millard P, Grelet G. 2010 Nitrogen storage and remobilization by trees: ecophysiological relevance in a changing world. Tree Physiology 30:1083–1095. 20551251 10.1093/treephys/tpq042

[plx007-B28] Millard P, Hester A, Wendler R, Baillie G. 2001 Interspecific defoliation responses of trees depend on sites of winter nitrogen storage. Functional Ecology 15:535–543.

[plx007-B30] Millard P, Proe MF. 1992 Storage and internal cycling of nitrogen in relation to seasonal growth of Sitka spruce. Tree Physiology 10:33–43. 14969873 10.1093/treephys/10.1.33

[plx007-B31] Millard P, Sommerkorn M, Grelet G-A. 2007 Environmental change and carbon limitation in trees: a biochemical, ecophysiological and ecosystem appraisal. New Phytologist 175:11–28. 17547663 10.1111/j.1469-8137.2007.02079.x

[plx007-B32] Miller HG. 1984 Dynamics of nutrient cycling in plantation ecosystems Nutrition of plantation forests. London: Academic Press, 53–78.

[plx007-B33] Miller-Pierce MR, Orwig DA, Preisser EL. 2010 Effects of hemlock woolly adelgid and elongate hemlock scale on Eastern hemlock growth and foliar chemistry. Environmental Entomology 39:513–519. 20388282 10.1603/EN09298

[plx007-B34] Mladenoff DJ, Stearns F. 1993 Eastern hemlock regeneration and deer browsing in the Northern Great Lakes Region: A re-examination and model simulation. Conservation Biology 7:889–900.

[plx007-B35] Nadelhoffer KJ, Emmett BA, Gundersen P, Kjønaas OJ, Koopmans CJ, Schleppi P, Tietema A, Wright RF. 1999 Nitrogen deposition makes a minor contribution to carbon sequestration in temperate forests. Nature 398:145–148.

[plx007-B36] Nambiar EKS, Fife DN. 1987 Growth and nutrient retranslocation in needles of Radiata pine in relation to nitrogen supply. Annals of Botany 60:147–156.

[plx007-B37] Näsholm T, Ericsson A. 1990 Seasonal changes in amino acids, protein and total nitrogen in needles of fertilized Scots pine trees. Tree Physiology 6:267–281. 14972938 10.1093/treephys/6.3.267

[plx007-B38] Paradis AF. 2011. Population dynamics of the hemlock woolly adelgid, University of Massachusetts Amherst, (Hemiptera: Adelgidae).

[plx007-B39] Preisser EL, Elkinton JS. 2008 Exploitative competition between invasive herbivores benefits a native host plant. Ecology 89:2671–2677. 18959304 10.1890/08-0299.1

[plx007-B40] Rapp M, Leclerc MC, Lossaint P. 1979 The nitrogen economy in a *Pinus pinea* L. stand. Forest Ecology and Management 2:221–231.

[plx007-B41] Raupp MJ, Denno RF. 1983 Leaf age as a predictor of herbivore distribution and abundance Variable plants and herbivores in natural and managed systems. New York: Academic Press, 91–124.

[plx007-B42] R Core Team. 2015 R: A language and environment for statistical computing. R Foundation for Statistical Computing, Vienna, Austria. URL https://www.R-project.org.

[plx007-B43] Reich PB, Grigal DF, Aber JD, Gower ST. 1997 Nitrogen mineralization and productivity in 50 hardwood and conifer stands on diverse Soils. Ecology 78:335–347.

[plx007-B44] Rennenberg H, Dannenmann M, Gessler A, Kreuzwieser J, Simon J, Papen H. 2009 Nitrogen balance in forest soils: nutritional limitation of plants under climate change stresses. Plant Biology 11:4–23. 19778364 10.1111/j.1438-8677.2009.00241.x

[plx007-B45] Schowalter TD, Webb JW, Crossley DA. 1981 Community structure and nutrient content of canopy arthropods in clearcut and uncut forest ecosystems. Ecology 62:1010–1019.

[plx007-B46] Soltis NE, Gómez S, Gonda-King L, Preisser EL, Orians CM. 2015 Contrasting effects of two exotic invasive hemipterans on whole-plant resource allocation in a declining conifer. Entomologia Experimentalis Et Applicata 157:86–97.

[plx007-B47] Souto D, Luther T, Chianese B. 1996 Past and current status of HWA in eastern and Carolina hemlock stands. *Proceedings of the First Hemlock Woolly Adelgid Review* Charlottesville, VA, 9–15.

[plx007-B48] Stockeler JH, Strothmann RO, Krefting LW. 1957 Effect of deer browsing on reproduction in the northern hardwood-hemlock type in northeastern Wisconsion. Journal of Wildlife Management 21:75–80.

[plx007-B49] Thomas RQ, Canham CD, Weathers KC, Goodale CL. 2010 Increased tree carbon storage in response to nitrogen deposition in the US. Nature Geoscience 3:13–17.

[plx007-B50] Tripler CE, Kaushal SS, Likens GE, Todd Walter M. 2006 Patterns in potassium dynamics in forest ecosystems. Ecology Letters 9:451–466. 16623731 10.1111/j.1461-0248.2006.00891.x

[plx007-B51] Ward JS, Montgomery ME, Cheah C. a S-J, Onken BP, Cowles RS. 2004 Eastern hemlock forests : Guidelines to minimize the impacts of hemlock woolly adelgid. *NA-TP-03–04*, *USDA Forest Service, Northeastern Area State and Private Forestry, Morgantown, WV*: 32.

[plx007-B52] Young RF, Shields KS, Berlyn GP. 1995 Hemlock woolly adelgid (Homoptera: Adelgidae): stylet bundle insertion and feeding sites. Annals of the Entomological Society of America 88:827–835.

[plx007-B53] Zvereva EL, Lanta V, Kozlov MV. 2010 Effects of sap-feeding insect herbivores on growth and reproduction of woody plants: a meta-analysis of experimental studies. Oecologia 163:949–960. 20397030 10.1007/s00442-010-1633-1

