# Supplementary material for: Seasonal variation in effects of herbivory on foliar nitrogen of a threatened conifer
Source: AoB Plants. 2017 Feb 28;9(2):plx007. doi: 10.1093/aobpla/plx007 (PMC5391718; doi:10.1093/aobpla/plx007)
Supplement: Supplementary Data [file plx007_Supp.docx]

**Table S1.** Analysis of variance (ANOVA) tables for effects of *Adelges tsugae* (HWA) on N(%), C:N ratio, and protein concentrations of mature (2011 growth) and young (2012 growth) *Tsuga canadensis* tissues from September to April.

| Response variable  Factor | N (%)^§^  *F*  Factor | *P* | C:N^†^  *F* | *P* |  | Protein  *F* | *P* |
| --- | --- | --- | --- | --- | --- | --- | --- |
| Treatment | 1.64 | 0.06 | 1.72 | 0.06 | | 0.04 | 0.83 |
| Tissue | 1412.69 | < 0.0001 | 1850.77 | < 0.0001 | | 157.67 | < 0.0001 |
| Tissue Age | 359.24 | < 0.0001 | 387.88 | < 0.0001 | | 3.27 | 0.07 |
| Time | 75.99 | < 0.0001 | 11.58 | < 0.001 | | 28.28 | < 0.0001 |
| Treatment x Tissue | 0.08 | 0.92 | 0.67 | 0.42 | | 0.003 | 0.96 |
| Treatment x Tissue Age | 9.98 | 0.001 | 6.35 | 0.012 | | 5.63 | 0.02 |
| Treatment x Time | 4.28 | 0.36 | 8.01 | 0.005 | | 0.16 | 0.93 |
| Tissue x Tissue Age | 16.45 | < 0.0001 | 34.27 | < 0.0001 | | 6.47 | 0.01 |
| Tissue x Time | 211.22 | < 0.0001 | 227.22 | < 0.0001 | | 18.48 | < 0.0001 |
| Tissue Age x Time | 10.46 | 0.001 | 10.56 | 0.001 | | 3.06 | 0.03 |
| Treatment x Tissue x Tissue Age | 3.02 | 0.08 | 1.96 | 0.16 | | 0.22 | 0.64 |
| Treatment x Tissue x Time | 0.02 | 0.90 | 1.27 | 0.26 | | 0.39 | 0.76 |
| Treatment x Tissue Age x Time | 0.03 | 0.87 | 0.46 | 0.50 | | 0.93 | 0.43 |
| Tissue x Tissue Age x Time | 5.06 | 0.02 | 1.77 | 0.18 | | 0.60 | 0.61 |
| Treatment x Tissue x Tissue Age x Time | 0.54 | 0.46 | 0.06 | 0.80 | | 0.28 | 0.84 |

^§^Square root-transformed for analysis

^†^Log-transformed for analysis

**Table S2.** Analysis of variance (ANOVA) tables for effects of *Adelges tsugae* (HWA) on N(%), C:N ratio, and protein concentrations of new *Tsuga canadensis* tissues produced during the 2013 season.

| Response variable  Factor | N (%)^§^  *F*  Factor | *P* | C:N^†^  *F* | *P* |  | Protein  *F* | *P* |
| --- | --- | --- | --- | --- | --- | --- | --- |
| Treatment | 3.80 | 0.06 | 4.89 | 0.04 | | 0.07 | 0.80 |
| Tissue | 344.83 | < 0.0001 | 410.11 | < 0.0001 | | 78.51 | < 0.0001 |
| Tissue Age | 181.68 | < 0.0001 | 234.53 | < 0.0001 | | 5.73 | 0.02 |
| Treatment x Tissue | 0.17 | 0.68 | 0.83 | 0.37 | | 1.19 | 0.28 |
| Treatment x Tissue Age | 0.01 | 0.92 | 0.50 | 0.48 | | 0.81 | 0.37 |
| Tissue x Tissue Age | 11.45 | 0.001 | 25.26 | < 0.0001 | | 14.66 | < 0.001 |
| Treatment x Tissue x Tissue Age | 0.83 | 0.36 | 0.56 | 0.46 | | 0.83 | 0.37 |

^§^Square root-transformed for analysis

^†^Log-transformed for analysis
